# Copper Oxide Nanoparticle-Induced Acute Inflammatory Response and Injury in Murine Lung Is Ameliorated by Synthetic Secoisolariciresinol Diglucoside (LGM2605)

**DOI:** 10.3390/ijms22179477

**Published:** 2021-08-31

**Authors:** Ralph A. Pietrofesa, Kyewon Park, Om P. Mishra, Darrah Johnson-McDaniel, Jacob W. Myerson, Vladimir V. Shuvaev, Evguenia Arguiri, Shampa Chatterjee, Ganesh S. Moorthy, Athena Zuppa, Wei-Ting Hwang, Melpo Christofidou-Solomidou

**Affiliations:** 1 Department of Medicine, Division of Pulmonary, Allergy, and Critical Care Medicine, Perelman School of Medicine, University of Pennsylvania, Philadelphia, PA 19104, USA; ralphp@pennmedicine.upenn.edu (R.A.P.); kyewpark@pennmedicine.upenn.edu (K.P.); Omprakash.Mishra@pennmedicine.upenn.edu (O.P.M.); evguenia@pennmedicine.upenn.edu (E.A.); 2 Cornell Center for Materials Research, Cornell University, Ithaca, NY 14850, USA; dj378@cornell.edu; 3 Department of Pharmacology, Perelman School of Medicine, University of Pennsylvania, Philadelphia, PA 19104, USA; myerson@pennmedicine.upenn.edu (J.W.M.); shuvaevv@pennmedicine.upenn.edu (V.V.S.); 4 Department of Physiology, Perelman School of Medicine, University of Pennsylvania, Philadelphia, PA 19104, USA; shampac@pennmedicine.upenn.edu; 5 Department of Anesthesiology and Critical Care Medicine, Children’s Hospital of Philadelphia, Philadelphia, PA 19104, USA; moorthyg@chop.edu (G.S.M.); zuppa@chop.edu (A.Z.); 6 Department of Biostatistics, Epidemiology, and Informatics, Perelman School of Medicine, University of Pennsylvania, Philadelphia, PA 19104, USA; whwang@pennmedicine.upenn.edu

**Keywords:** active chlorine species, chlorination damage, copper oxide, inflammation, LGM2605, myeloperoxidase, nanoparticles, oxidative stress, ROS, secoisolariciresinol diglucoside

## Abstract

Metal-oxide nanoparticles (MO-NPs), such as the highly bioreactive copper-based nanoparticles (CuO-NPs), are widely used in manufacturing of hundreds of commercial products. Epidemiological studies correlated levels of nanoparticles in ambient air with a significant increase in lung disease. CuO-NPs, specifically, were among the most potent in a set of metal-oxides and carbons studied in parallel regarding DNA damage and cytotoxicity. Despite advances in nanotoxicology research and the characterization of their toxicity, the exact mechanism(s) of toxicity are yet to be defined. We identified chlorination toxicity as a damaging consequence of inflammation and myeloperoxidase (MPO) activation, resulting in macromolecular damage and cell damage/death. We hypothesized that the inhalation of CuO-NPs elicits an inflammatory response resulting in chlorination damage in cells and lung tissues. We further tested the protective action of LGM2605, a synthetic small molecule with known scavenging properties for reactive oxygen species (ROS), but most importantly, for active chlorine species (ACS) and an inhibitor of MPO. CuO-NPs (15 µg/bolus) were instilled intranasally in mice and the kinetics of the inflammatory response in lungs was evaluated 1, 3, and 7 days later. Evaluation of the protective action of LGM2605 was performed at 24 h post-challenge, which was selected as the peak acute inflammatory response to CuO-NP. LGM2605 was given daily via gavage to mice starting 2 days prior to the time of the insult (100 mg/kg). CuO-NPs induced a significant inflammatory influx, inflammasome-relevant cytokine release, and chlorination damage in mouse lungs, which was mitigated by the action of LGM2605. Preventive action of LGM2605 ameliorated the adverse effects of CuO-NP in lung.

## 1. Introduction

Nanotechnology has found usefulness in diverse applications, ranging from biomedical applications to food packaging and electronics [1]. Excess use of such nanomaterial, however, results in increased toxicity to humans and the environment [2]. Metal oxide nanoparticles (MO-NPs), generated in facilities where antimicrobial products, catalysts, and other technologies are being generated [3,4], can be inhaled as copper fumes and, thus, enter the respiratory system causing adverse health effects. These range from acute lung injury (ALI) to more chronic responses, such as lung fibrosis and even malignancy [5,6,7].

Copper-oxide nanoparticles (CuO-NPs) are an important class of materials with applications as catalysts, conductive inks, and antimicrobial agents [3,8,9,10,11]. Environmental and safety issues are particularly important for copper-based nanomaterials because of their potential large-scale use and their high redox activity, and toxicity reported from in vitro studies [6,12,13]. Despite advances in nanotoxicology research, the exact mechanism of CuO-NP toxicity has not been elucidated. Since exposure occurs mostly through inhalation of such nanoparticles in dust or fumes, we have focused our studies on lung toxicity to test our hypothesis [14,15,16,17,18,19,20].

Toxicological research on CuO-NPs revealed significant toxicity relative to other common nanomaterials [6,12,21,22,23,24,25,26]. In fact, CuO-NPs were the most potent regarding DNA damage and cytotoxicity in a set of metal oxides and carbons studied in parallel [10]. Additionally, CuO-NP exposure resulted in oxidative stress-induced cytotoxicity in airway epithelial (HEp-2) cells [13] and aggravated the asthmatic phenotype in mice [16]. There is a consensus that the cytotoxicity and genotoxicity associated with CuO-NPs is due, at least in part, to reactive oxygen species (ROS) generation and activation of oxidative pathways [27], yet effective antioxidant protective strategies have shown limited benefit [28]. Our interest in this class of harmful agents (chlorine species) resulted from studies where we identified significant generation of active chlorine species (ACS) in response to stressors, such as radiation [29,30], or in connection with inflammation and release of damaging chlorine species by the myeloperoxidase (MPO) in granulocytes (macrophages and neutrophils) [31]. In the present studies, we aimed to test the hypothesis that chlorination damage is a potential mechanism of CuO-NP-induced toxicity in tissues.

Copper has been used since ancient times in various applications and scientists have exploited its means of exposure and consequences to living organisms [32]. The unique property of CuO-NPs having a high surface to volume ratio has increased the range of application in diverse products. CuO-NPs are widely used in industrial applications, such as semiconductor devices, gas sensor, batteries, solar energy converter, microelectronics, heat transfer fluids, and consumer products. Since acute toxicity of CuO-NPs has been reported; therefore, human and environmental health may be at a high risk. Their frequent use can also contaminate ecosystems. Therefore, the mechanism(s) of toxicity of CuO-NPs needs to be thoroughly understood.

We characterized an acute mouse exposure model of harmful nanoparticle material deposition in lung, which will facilitate the evaluation of mechanistic hypotheses of tissue toxicity. We will test whether CuO-NP-induced chlorination damage is linked to tissue toxicity. Our hypothesis linking inflammation, chlorination damage, and cell death induced by CuO-NP is paradigm-shifting since, thus far, only oxidative and nitrosative damage has been linked to metal oxide nanoparticle damage.

LGM2605, the synthetic lignan secoisolariciresinol diglucoside (SDG), is a potent antioxidant and free radical scavenger with anti-inflammatory properties [29,30,33]. We have shown that LGM2605 is an ACS scavenger and inhibitor of myeloperoxidase, the enzyme in inflammatory cells that generates HOCl [31]. In addition, we have previously identified several key pathways through which LGM2605 is able to prevent DNA damage and reduce tissue damage and inflammation [34,35,36,37,38]. In the current study, we established the kinetics of the inflammatory response to CuO-NP challenge in murine lungs and we evaluated the therapeutic role of LGM2605 treatment in ameliorating inflammation and chlorination damage of lung tissues. Chlorination damage resulting from CuO-NP exposure of lungs detected by ultra-performance liquid chromatography (UPLC)-tandem mass spectrometry (MS-MS) has never been shown.

## 2. Results

In this study, we demonstrated an acute inflammatory response in murine lungs following exposure to CuO-NPs and, subsequently, evaluated the treatment effects of synthetic secoisolariciresinol diglucoside, LGM2605, on chlorination toxicity and select inflammatory outcomes of CuO-NP exposure in lung tissues.

### 2.1. Characterization of CuO-NPs and Carbon Black M120

**Figure 1:**
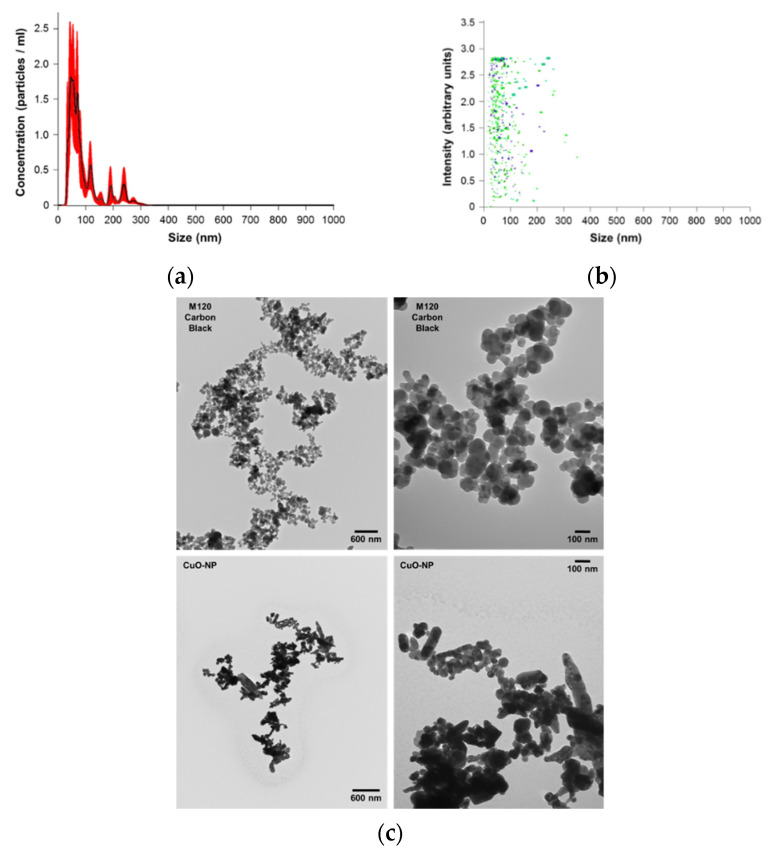

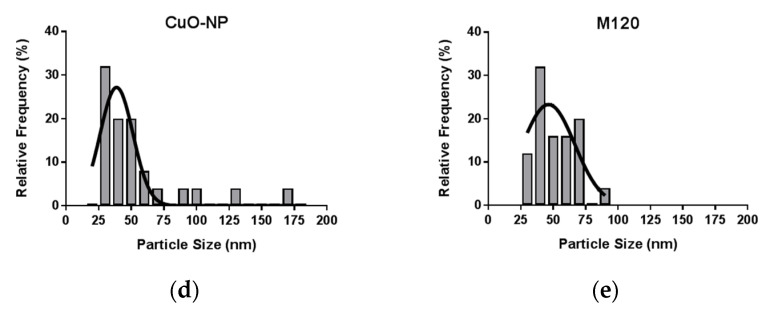
Characterization of CuO-NP and M120 nanoparticles. (**a**,**b**) show the effective diameter of nanomaterial/particles as measured by nanoparticle tracking analysis (NTA); (**c**) shows the transmission electron microscopy (TEM) characterization of size/shape of the nanoparticles; (**d**,**e**) show the plotted histograms displaying the variability in particle size for CuO-NP and M120.

The effective diameter of nanomaterials/particles was measured by dynamic light scattering (DLS) and nanoparticle tracking analysis (NTA). Figure 1a,b show scattering mode NTA determination of the size distribution profile of CuO-NP at 1:20 vol:vol dilution of delivered nanoparticle stock. A five-measurement average determined a nanoparticle diameter of 88.2 nm ± 10.3 nm and a particle concentration of 1.06 × 10^7^ ± 8.043 × 10^5^ particles per ml, indicating that ~2 × 10^7^ CuO-NPs were administered per mouse. Figure 1c shows the TEM characterization of size/shape of CuO-NP probes and control carbon black M120 [39]. The results confirm that both CuO-NP (Figure 1d) and M120 (Figure 1e) preparations were comprised of a mixture of elongated and spherical particles of comparable size.

### 2.2. Kinetics of CuO-NP-Induced Acute Lung Injury in Mice

**Figure 2:**
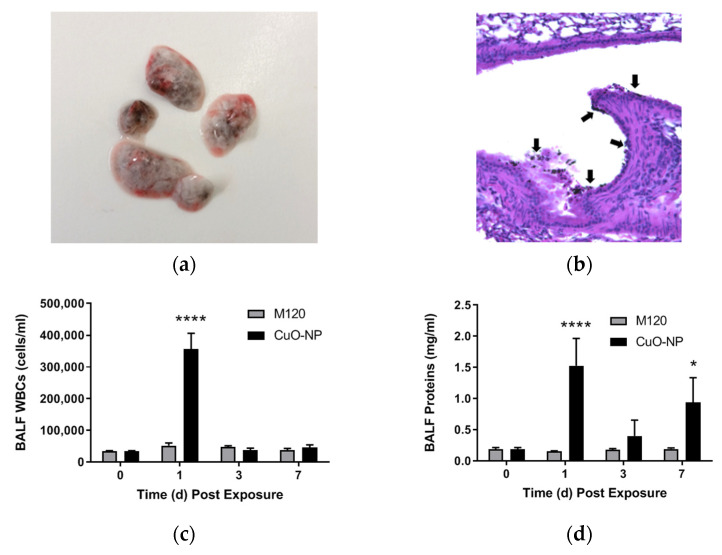

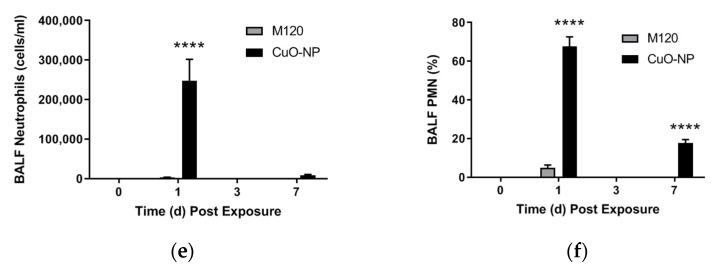
Kinetics of CuO-NP-induced Lung Injury in Mice. (**a**) representative clinical image of murine lungs extracted 4 h following intranasal administration of 15 µg CuO-NPs; (**b**) representative H & E section of a mouse lung 1 h following CuO-NP exposure (**c**) kinetics of BALF WBCs; (**d**) kinetics of BALF protein levels; (**e**) kinetics of BALF PMNs; (**f**) kinetics of BALF percent PMN determined 0, 1, 3, and 7 days post-CuO-NP or M120 exposure. All data are presented as the mean ± the standard error of the mean (*n* = 3–7 per group per time point). Asterisks indicate a statistically significant difference (* *p* < 0.05 and **** *p* < 0.0001) from their respective time 0.

The kinetics of CuO-NP-induced acute lung injury in mice was determined by intranasal instillation of CuO-NPs as compared to inert, carbon black M120 control [40], which is weakly bioreactive and was used as a non-toxic reference particle of comparable size to the CuO-NP [41]. The particle load in lungs instilled intranasally with the nanoparticles are visible macroscopically by clinical evaluation of the lungs 4 h following instillation (Figure 2a). Figure 2b shows an H & E section of a mouse lung 1 h post-CuO-NP instillation and shows an even distribution of the nanoparticles along the epithelial lining of the airways. Lungs were evaluated on days 0, 1, 3, and 7 following intranasal challenge to either agent. As anticipated, M120 did not induce a statistically significant inflammatory response or lung injury, as assessed by the bronchoalveolar lavage fluid (BALF) content of WBCs (Figure 2c) and PMN (Figure 2e,f), or BALF protein content (Figure 2d), respectively. CuO-NP instillation, however, induced a robust response with significant (*p* < 0.0001) inflammatory influx and lung injury at day 1, which dissipated by day 7. Interestingly, lung injury persisted for 7 days post-challenge. Thus, we selected 24 h post-CuO-NP challenge to evaluate the protective effects of LGM2605 as the time point that elicits a significant inflammatory response.

### 2.3. LGM2605 Treatment Reduces Lung Injury and Inflammation Following CuO-NP Exposure

**Figure 3:**
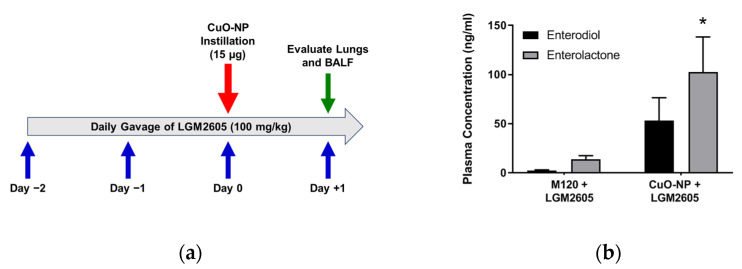
Experimental Scheme and Plasma Levels of LGM2605 (Synthetic SDG) Metabolites in Mice. (**a**) displays the experimental scheme, where LGM2605 administration was initiated 2 days prior to CuO-NP exposure; (**b**) shows the plasma levels of LGM2605 (synthetic SDG) metabolites, the mammalian lignans enterodiol (ED) and enterolactone (EL), in mice exposed to M120 or CuO-NP (*n* = 5 per group) using LC-MS/MS. Data are presented as the mean ± the standard error of the mean. Asterisks indicate a statistically significant difference (* *p* < 0.05) from M120 + LGM2605.

To determine the effects of LGM2605 in abrogating CuO-NP-induced lung inflammation, we administered the agent daily via gavage at 100 mg/kg dose, starting 2 days prior to CuO-NP challenge; based on previous experience in other rodent inflammatory models [42] and continued until mice were euthanized and lungs evaluated at 24 h post-challenge (see experimental plan in Figure 3a). Importantly, the presence of SDG (LGM2605) mammalian metabolites enterodiol (ED) and enterolactone (EL) were confirmed in the systemic circulation using LC-MS/MS (Figure 3b).

**Figure 4:**
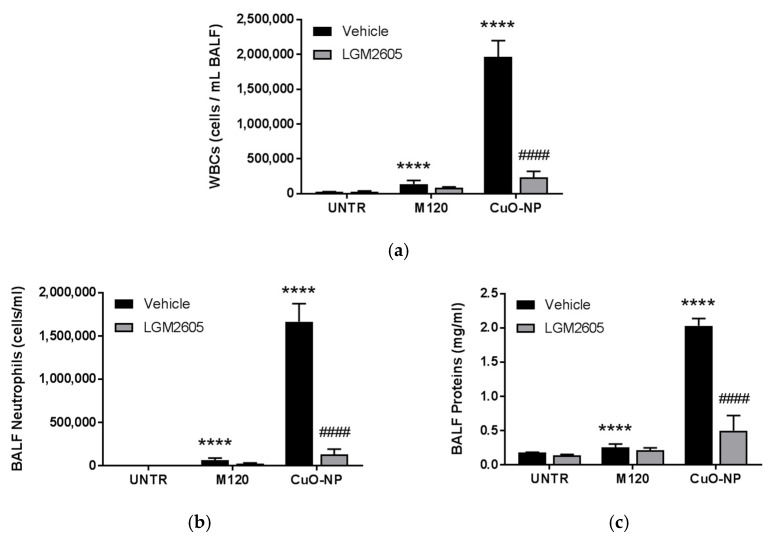
Prevention of CuO-NP-induced Acute Lung Injury in Mice by LGM2605. (**a**) BALF WBCs; (**b**) BALF PMNs; (**c**) BALF protein levels determined at 24 h following instillation of inert carbon control (M120) or 15 µg CuO-NPs. All data are presented as the mean ± the standard error of the mean (*n* = 2–6 per treatment and exposure group). Asterisks indicate a statistically significant difference from their respective unexposed, control group (UNTR) (**** *p* < 0.0001) and # indicates a statistically significant difference from Vehicle (#### *p* < 0.0001).

Bronchoalveolar lavage (BAL) was performed and fluid evaluated from lungs treated with M120 control or CuO-NP to determine the immune response and extent of lung injury. As anticipated, CuO-NPs induced a significant immune response (*p* < 0.0001) as compared to unexposed control mice (Figure 4a,b), as well as lung injury (*p* < 0.0001) by evaluating plasma protein leak into the BALF (Figure 4c). Importantly, LGM2605 attenuated both inflammation (Figure 4a,b) and injury (Figure 4c) significantly as compared to CuO-NP (*p* < 0.0001).

**Figure 5:**
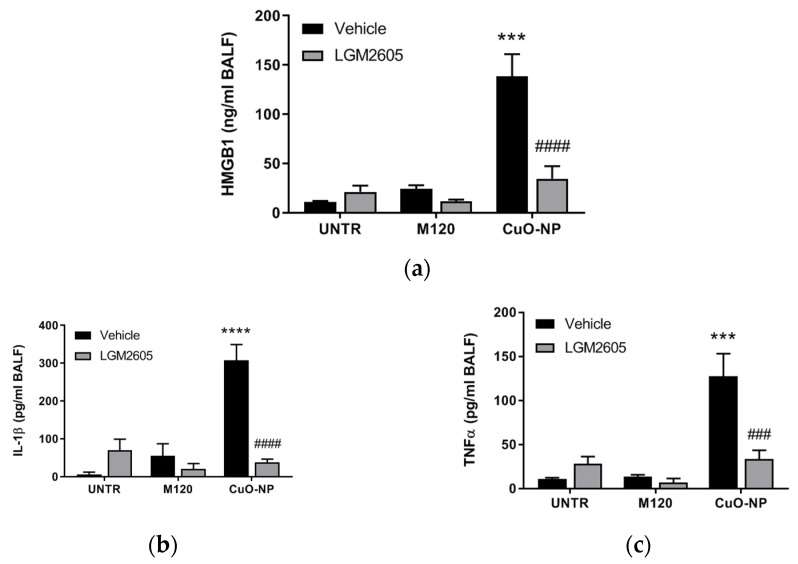
LGM2605 reduces lung levels of CuO-NP-induced inflammasome cytokines. (**a**) BALF HMGB1; (**b**) BALF IL-1β; (**c**) BALF TNFα determined at 24 h following instillation of inert carbon control (M120) or 15 µg CuO-NPs. All data are presented as the mean ± the standard error of the mean (*n* = 3–7 per treatment and exposure group). Asterisks indicate a statistically significant difference from their respective unexposed, control group (UNTR) (*** *p* < 0.001 and **** *p* < 0.0001) and # indicates a statistically significant difference from Vehicle (### *p* < 0.001 and #### *p* < 0.0001).

Levels of the proinflammatory cytokines, HMGB1 (Figure 5a), IL-1β (Figure 5b), and TNFα (Figure 5c), followed a similar profile, where levels were significantly elevated among mice exposed to CuO-NP, but attenuated by the presence of LGM2605. Importantly, no statistically significant difference was observed among any of the exposure groups (UNTR, M120, and CuO-NP) for mice treated with LGM2605.

### 2.4. LGM2605 Treatment Reduces Protein Chlorination in Murine Lung Following CuO-NP Exposure

**Figure 6:**
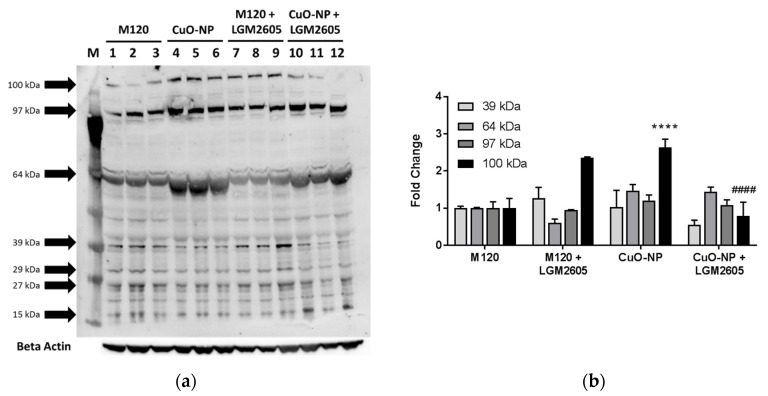
LGM2605 treatment reduces CuO-NP-induced increase in protein chlorination in murine lung. (**a**) Western blot analysis of chlorinated lung proteins (M, marker; lanes 1–3, M120; lanes 4–6, CuO-NP; lanes 7–9, M120 + LGM2605; lanes 10–12, CuO-NP + LGM2605); (**b**) densitometric analysis of chlorinated proteins from murine lung homogenates exposed to M120 or CuO-NP and treated with or without LGM2605. All data are presented as the mean ± the standard error of the mean. Asterisks indicate a statistically significant difference from M120 (**** *p* < 0.0001) and # indicates a statistically significant difference from CuO-NP (#### *p* < 0.0001).

While oxidative and nitrosative stress in tissues following exposure to metal oxide nanoparticles has been explored [43], chlorination stress resulting from the influx of MPO-rich neutrophils and macrophages has never been shown. Using a commercially available anti-chlorotyrosine antibody, we probed lung proteins using Western blot analysis (Figure 6a). This semiquantitative evaluation of lung chlorination indicated a large number of chlorinated proteins in response to all treatments (15, 27, 29, 39, 64, 97, and 100 kDa). LGM2605 had a moderate effect in mitigating chlorination intensity as evidenced by Western blot analysis (Figure 6b).

**Figure 7:**
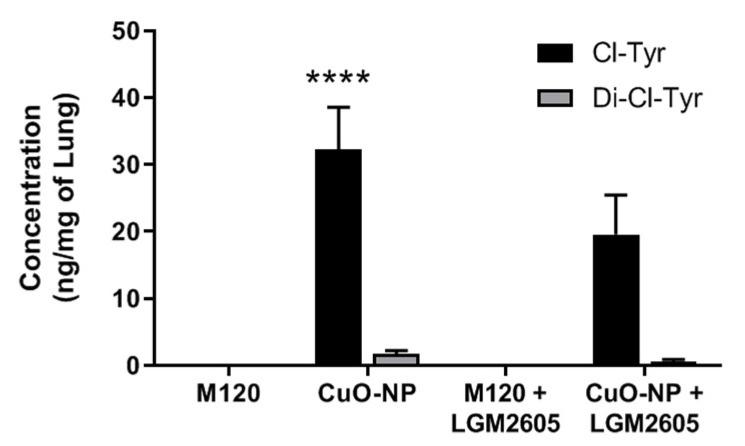
CuO-NP-induced protein chlorination in murine lung. Determination of mono-chlorotyrosine and di-chlorotyrosine in murine lung as determined by LC-MS/MS. All data are presented as the mean ± the standard error of the mean. Asterisks indicate a statistically significant difference from M120 (**** *p* < 0.0001).

To quantitatively probe the lung tissues for chlorinated proteins, we developed analytical tools to identify lung levels of 3-chlorotyrosine and 3,5-dichlorotyrosine based on an assay developed by Crow et al. [44] (Figure 7). Exposure to CuO-NPs led to a significant increase (*p* < 0.05) in mono-chlorotyrosine, an index of chlorination toxicity, in mouse lung while the level of di-chlorotyrosine did not significantly increase following treatment with CuO-NP. Treatment with M120 did not alter protein chlorination levels as compared to untreated controls (Figure 7).

### 2.5. CuO-NPs Induce ACS Generation by Activation of Myeoloperoxidase (MPO)

**Figure 8:**
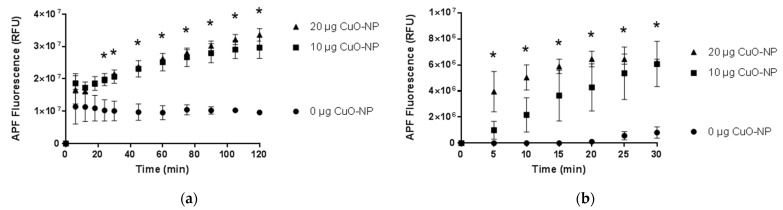
CuO-NPs activate MPO to generate ACS. (**a**) MPO-dependent ACS generation by CuO-NPs using purified MPO enzyme; (**b**) MPO-dependent ACS generation by CuO-NPs using bone marrow-derived mouse neutrophils. Data are presented as the mean ± the standard error of the mean. Asterisks indicate a statistically significant difference (* *p* < 0.05) from 0 µg CuO-NP.

We confirmed fluorometrically that CuO-NPs activate MPO to generate ACS, using either purified MPO enzyme (Figure 8a) or bone marrow-derived mouse neutrophils (Figure 8b) incubated with increasing concentrations of CuO-NP (10 and 20 µg CuO-NP). CuO-NPs induced a statistically significant increase in MPO-dependent ACS as determined by APF fluorescence.

### 2.6. LGM2605 Treatment Mitigates CuO-NP-Induced Acute Lung Inflammation and Injury When Administered Post-CuO-NP Exposure

**Figure 9:**
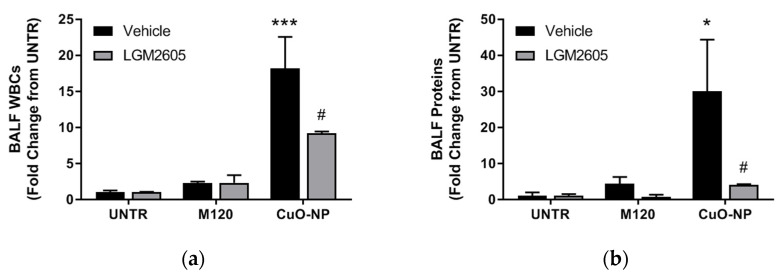
Mitigation of CuO-NP-induced acute lung injury by LGM2605. (**a**) BALF WBCs; and (**b**) BALF protein levels determined at 24 h following instillation of inert carbon control (M120) or 15 µg CuO-NPs. LGM2605 (100 mg/kg) was administered 1 h post-CuO-NP exposure. All data are presented as the mean ± the standard error of the mean (*n* = 3–5 per treatment and exposure group). Asterisks indicate a statistically significant difference from their respective unexposed, control group (UNTR) (* *p* < 0.05 and *** *p* < 0.001) and # indicates a statistically significant difference from vehicle (# *p* < 0.05).

In preliminary, proof of concept studies to determine the ability of LGM2605 to mitigate CuO-NP-induced lung inflammation and injury when administered therapeutically (following CuO-NP insult), animals were exposed to CuO-NPs or M120 and administered 100 mg/kg LGM2605 subcutaneously 1 h post-CuO-NP exposure. As previously observed, CuO-NP exposure led to a statistically significant increase in both BALF WBCs (Figure 9a) and proteins (Figure 9b). Similar to previous observations where LGM2605 was administered prior to nanoparticle exposure, treatment with LGM2605 after CuO-NP exposure led to a significant decrease in both BALF WBCs and proteins.

## 3. Discussion

Exposure to nanoparticles has several well-documented health effects leading to neoplastic, fibrotic, and immune outcomes, which appear to be largely rooted in early inflammatory responses to nanoparticles. While the long-term outcomes are severe and refractory to treatment, it is possible that treatments could ameliorate disease by modulating steps within the early inflammatory cascade. Here, we examined the early inflammatory in vivo effects of lung exposure to CuO-nanoparticles, and evaluated the potential for LGM2605 to block these responses. Additionally, we examined protein chlorination toxicity responses to intranasal CuO-NP exposure, as well as to LGM2605 treatment, given alone or in combination with CuO-NP exposure. To our knowledge, this is the first study to examine CuO-NPs chlorination toxicity and the abrogation/mitigation by LGM2605. Lastly, LGM2605 significantly reduced CuO-NP inflammation and lung damage when administered preventively or therapeutically.

LGM2605, synthetic SDG, is a potent antioxidant and free radical scavenger with anti-inflammatory properties [34,36,37,42,45]. We have shown that LGM2605 is a scavenger of active chlorine species (ACS) and inhibitor of myeloperoxidase (MPO), the enzyme in inflammatory cell that generates HOCl [29,30,31]. In addition, we have previously identified several key pathways through which LGM2605 is able to prevent DNA damage and reduce tissue damage and inflammation [36,46]. In the current study, LGM2605 treatment was initiated 2 days prior to a 15 µg bolus dose of CuO-NP, continued for a day, and immune responses assessed 1 day after the nanoparticle fiber exposure. Acute exposure to CuO-NP induced inflammation, characterized by an increase in lung protein levels, lung WBC accumulation, and an increase in lung neutrophils. LGM2605 significantly ameliorated lung proteins and WBC influx, specifically PMN. In addition, LGM2605 prevented the CuO-NP-induced increase in HMGB1 levels, indicating the amelioration of CuO-NP induced inflammatory response. Most importantly, CuO-NP exposure resulted in protein chlorination toxicity which was blocked by LGM2605. We have previously reported similar findings among Nf2^+/mut^ mice placed on an SDG-rich diet 7 days prior to 400 µg crocidolite asbestos exposure [46]. Given these outcomes, LGM2605 may be capable of reducing early inflammatory events induced by CuO-NP, which are thought to contribute to the progression of several CuO-NP-related diseases.

Neutrophils and macrophages produce many proinflammatory and fibrotic cytokines and are mediators of oxidative stress in tissues. Upon infection or injury, these cells migrate to sites of inflammation and have been implicated in the pathogenesis of a variety of asbestos-related diseases, including mesothelioma, lung fibrosis, and autoimmunity [47,48,49,50,51]. In this study, LGM2605 treatment blocked CuO-NP-induced recruitment of inflammatory cells to the lung. These results indicate that LGM2605 blocks the early pro-inflammatory responses associated with metal oxide toxicity. Furthermore, in feasibility studies, LGM2605 was able to mitigate the CuO-NP-induced increases in BALF WBCs and proteins when given 1 h post-nanoparticle exposure.

The mechanism of LGM2605 on the reduction of neutrophil recruitment was not examined within this study, but it may be related to the ability of drug to reduce local ROS/RNS and to block inflammasome activation, mechanisms previously described [35,36]. Specifically, CuO-NP exposure may cause oxidative stress in tissue that was alleviated with LGM2605 treatment [35]. Reduction of local free radicals would reduce local tissue damage, which may then reduce inflammatory responses and immune cell recruitment. We have shown that LGM2605 is a free radical scavenger and a potent antioxidant. Additionally, LGM2605 was also shown to block the activation of NLRP3 inflammasome in macrophages [36]. Inflammasome activation results in the release of the proinflammatory cytokines IL-1β and IL-18, which then contribute to innate cell recruitment and activation. Therefore, LGM2605 treatment may reduce innate immune cell influx by limiting early inflammatory events at the site of tissue injury by asbestos and asbestos-like fiber deposition, which may in turn have significant protective properties against the development of CuO-NP-induced diseases.

**Figure 10:**
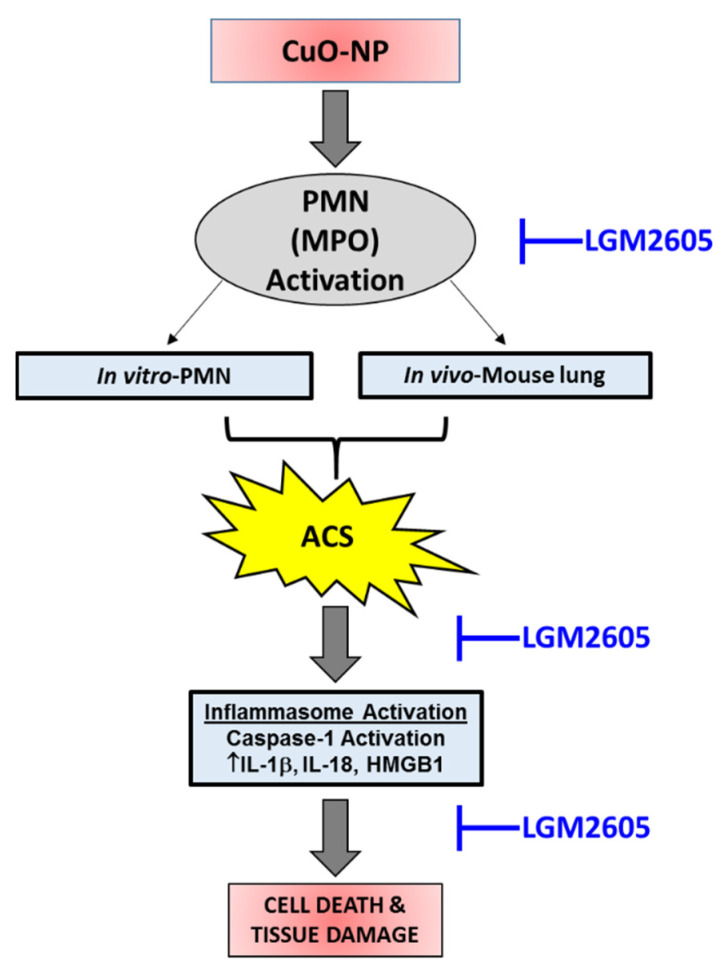
Proposed mechanism of CuO-NP-induced lung toxicity and LGM2605 protection. MPO-dependent, active chlorine species (ACS)-induced inflammasome activation is associated with lung toxicity following CuO-NP exposure. LGM2605 abrogates CuO-NP-induced lung toxicity by reducing ACS generation and inhibiting inflammasome activation.

An important part of this investigation was to examine the effect of CuO-NP on protein chlorination toxicity. Accumulating evidence points to metal oxide-induced inflammasome activation [52] in diverse cell types such as blood monocytes [53] or alveolar macrophages [54]. CuO-NPs are an important class of materials with applications as catalysts, conductive inks, and antimicrobial agents [3,8,9,10,11]. CuO-NPs, specifically, were among the most potent in a set of metal-oxides and carbons studied in parallel regarding DNA damage and cytotoxicity [10]. Despite advances in nanotoxicology research and characterization of their toxicity [55], the exact mechanism(s) of toxicity are yet to be defined [17,56]. There is a general consensus that the cytotoxicity and genotoxicity associated with copper-based nanoparticles is due to reactive oxygen species (ROS) generation and activation of oxidative pathways [27]. However, the cellular mechanisms driving inflammation by CuO-nanoparticles are yet to be elucidated. We pioneered the studies showing generation of active chlorine species (ACS) in response to stressors such as radiation [29,30] and asbestos or in connection with inflammation and activation of myeloperoxidase (MPO) in macrophages and neutrophils resulting in damage to macromolecules [31]. In this study, we examined the hypothesis that CuO-NP-induced inflammation and lung toxicity is mediated by ACS generated by CuO-NP-induced activation of MPO. The results show that CuO-NP exposure resulted in increased protein tyrosine chlorination. Myeloperoxidase (MPO) is the only enzyme that generates active HOCl species that can chlorinate proteins. CuO-NP-induced production of protein chlorotyrosines confirms the generation of HOCl as a consequence of MPO activation. The proposed mechanism of CuO-NP-induced lung toxicity, through ACS generation and inflammasome activation, and LGM2605 protection is presented in Figure 10.

**Figure 11:**
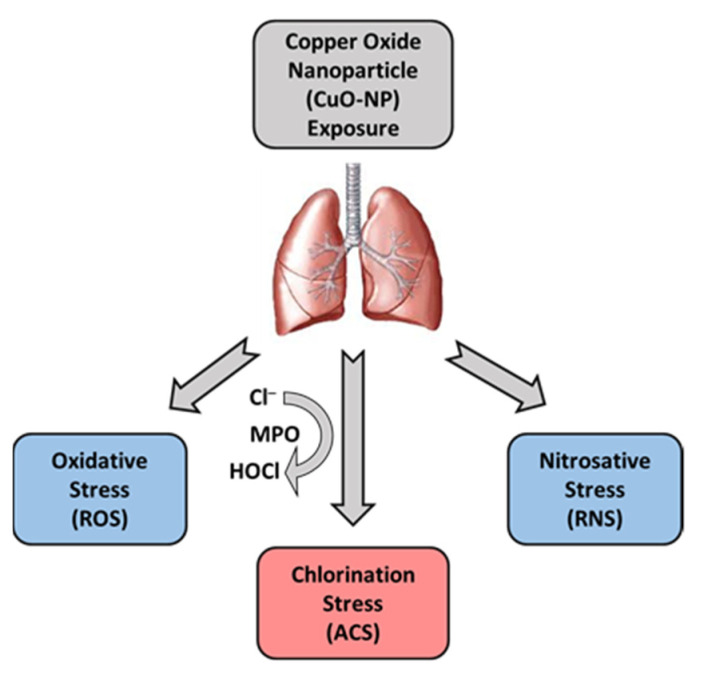
Schematic presentation of CuO-NP-induced lung inflammation and injury. CuO-NPs induce, in addition to nitrosative and oxidative stress in lung tissues, chlorination damage by the action of ACS generated by recruited PMN and macrophages.

As mentioned before, MPO is expressed in neutrophils, monocytes, and some tissue macrophages, and generates HOCl during inflammation and infection. MPO is a key factor in cardiovascular, neurodegenerative, inflammatory, and immune-mediated diseases and catalyzes the reaction between physiologically present Cl– and hydrogen peroxide (H_2_O_2_) to generate a potent oxidant, HOCl [57,58,59] (Figure 11). HOCl and its conjugate base ClO– oxidize amino acids, peptides, proteins, and lipids [60,61,62,63] and chlorinate nuclear bases in cellular DNA and RNA [60,64,65]. Based on protein tyrosine chlorination, we propose that CuO-NP exposure leads to activation of MPO that results in increased generation of HOCl and protein chlorination in CuO-NP exposed mice. The proposed mechanism of CuO-NP-induced cytotoxicity and cell death is presented in Figure 11. In addition, CuO-NP may activate inflammasomes leading to generation of proinflammatory cytokines that elicit proinflammatory response. We have recently shown that LGM2605 scavenges HOCl [29,30] and inhibits MPO in inflammatory cells [31]. In addition, LGM2605 prevents asbestos-induced inflammasome activation [36]. These appear to be the proposed mechanisms of LGM2605 preventive action on CuO-NP induced inflammation and tissue protein chlorination toxicity.

## 4. Materials and Methods

### 4.1. Nanoparticles

CuO-NPs with a primary particle size reported by the manufacturer of 50 nm were purchased from Nanostructured and Amorphous Materials, Inc. (Houston, TX, USA) and used without any further intentional modification. Monarch 120 (M120) specialty carbon black was purchased from Cabot Corporation (Boston, MA, USA).

### 4.2. Dynamic Light Scattering (DLS) and Nanoparticle Tracking Analysis (NTA) for Characterization of Nanoparticles

The effective diameter of nanomaterial/particles was measured in the laboratory of Dr. Vladimir Muzykantov (UPenn) by (1) DLS using a Zetasizer Nano ZSP (Malvern Instruments Ltd., Malvern, UK) with 173° backscatter and (2) NTA using a NanoSight NS300 (Malvern Instruments Ltd., Malvern, UK). The measurements by DLS were performed at 25 °C using 2 min as an equilibration time. The data were analyzed by the number of particles and showed two populations of particles: <100> nm (96%) and <500> nm (4%) for CuO-NP and <100> nm (96%) and <500> nm (4%) for M120 carbon black to confirm that both preparations are of equal size distribution. NanoSight single nanoparticle tracking analysis was performed in scattering mode to determine the size distribution profile and numerical concentration of CuO-NPs at 1:20 vol:vol dilution of delivered nanoparticle stock.

### 4.3. Transmission Electron Microscopy (TEM) Characterization of Size/Shape of Nanoparticle Probes

CuO-NP and control carbon black M120 suspensions were prepared in sterile water at concentrations used in animal studies [40]. Following ultrasonication on ice for 1 min, electron microscopy (EM) grids were prepared and viewed on an 80 kV JEOL JEM 1010 transmission electron microscope (JEOL USA, Peabody, MA, USA) at both 25,000× and 100,000× magnification (Electron Microscopy Resource Lab at the Perelman School of Medicine, University of Pennsylvania). The length and diameter distributions of all nanoparticle samples were obtained by image analysis of EM micrographs. Means and standard deviations were computed from at least one hundred single particle measurements using NIH ImageJ software (Fiji Version, National Institutes of Health, Bethesda, MD, USA). Analysis confirmed that both CuO-NP and M120 preparations were comprised of a mixture of elongated and spherical particles of comparable size.

### 4.4. Mouse Exposure to CuO-NP

Mice were obtained from Charles River (Wilmington, MA, USA) under animal proto-cols approved by the Institutional Animal Care and Use Committee (IACUC) of the University of Pennsylvania. Animals were housed in conventional cages under standardized conditions with controlled temperature and humidity and a 12:12 h day–night light cycle. Animals had access to water and standard mouse chow ad libitum. Our kinetic studies and proof of concept studies both used female C57BL/6 mice (*n* = 5 mice per group unless otherwise stated).

Methods for intranasal instillation have been previously described [66]. In brief, mice were placed in an induction chamber and lightly anesthetized with isoflurane. Once anesthetized, mice were removed from the induction chamber and placed in a supine position and 100 µL of instillate (total dose) delivered dropwise to the nares using a pipette.

CuO-NPs were suspended in sterile water and given intranasally. Inhalation studies are the gold standard for assessing the potential risks of exposure to air-borne particulates. Biological responses seen in the respiratory tract or other tissues after inhalation are routinely correlated with measures of external exposure (e.g., mg/m^3^) or inhaled dose (mg/kg) or more correctly as retained dose in lung tissues. We selected a bolus dose of 15 µg, which is within the range of 0–13.2 mg/m^3^ (2.67–57.87 µM)/exposure, based on established doses of inhalational exposure [17] and intranasally instilled NPs (2.5–5.0 mg/kg) [67]. The experimental protocol for LGM2605 treatment and CuO-NP exposure is shown in Figure 3a.

### 4.5. LGM2605 Treatment

LGM2605 was prepared as previously described [68]. Briefly, LGM2605 was synthesized from vanillin via secoisolariciresinol and a glucosyl donor (perbenzoyl-protected trichloroacetimidate under the influence of TMSOTf) through a concise route that involved chromatographic separation of diastereomeric diglucoside derivatives (Chemveda Life Sciences, Inc., Hyderabad, India). Lyophylized samples of LGM2605 at 100 mg/vial were reconstituted daily with sterile water. Oral gavages (100 mg/kg) were performed daily using sterile angled gavage needles. Mice were weighed every day LGM2605 was administered, starting 2 days prior to CuO-NP exposure and continuing for 1 day after exposure (see Figure 3a). In the feasibility study, the mitigating effects of LGM2605 were evaluated by administering drug (100 mg/kg) subcutaneously 1 h post-CuO-NP exposure.

### 4.6. Analytical Evaluation of Lignan Content in Murine Plasma Samples

Plant lignans, such as the lignan SDG, are metabolized by intestinal bacteria to the mammalian lignans, enterodiol (ED) and enterolactone (EL), that can be readily detected in plasma. Circulating plasma levels of ED and EL at the time of study termination were determined by liquid chromatography/tandem mass spectrometry (LC-MS/MS) as described earlier [69,70,71] using commercially available standards in 95% purity (Chromadex, Inc., Santa Ana, CA, USA). Plasma SDG metabolite levels were evaluated in 3 randomly selected mice per cohort.

### 4.7. Evaluation of Lung Injury

Mice were euthanized using an overdose of ketamine (160 mg/kg) and xylazine (25 mg/kg) at 1 and 7 days from challenge with CuO-NP. Bronchoalveolar lavage (BAL) was then performed as described previously [72]. Briefly, BAL was performed through a 20-gauge angiocatheter (BD Pharmingen, San Diego, CA, USA), with the intratracheal instillation of 1 mL of phosphate-buffered saline (PBS) containing an antiprotease cocktail (MilliporeSigma, Burlington, MA, USA) and 5 mM EDTA given in 0.5 mL increments [69,70,73]. An aliquot was immediately separated to measure total leukocyte cell counts (cells/mL BALF) using a Coulter Cell and Particle Counter (Beckman Coulter, Miami, FL, USA). The remaining lavage fluid was centrifuged at 1200 rpm for 10 min and the cell-free supernatant was frozen at −80 °C for cytokine and protein analysis.

The amount of total protein in the BALF was assayed using the BCA Protein Assay Kit (Pierce, Rockford, IL) as per manufacturer’s instructions. Absorbance was read at 560 nm (MRX Microplate Reader, Dynatech Laboratories, Chantilly, VA, USA) and protein levels in mg/mL of BALF were calculated. The results are reported as fold change from untreated control.

### 4.8. Analytical Determination of Chlorotyrosine and 3,5-Dichlorotyrosine in Murine Lung

A sensitive ultra-performance liquid chromatography (UPLC)-tandem mass spectrometry (MS-MS) assay for 3-Chlorotyrosine (Cl-Tyr) and 3,5-Dichlorotyrosine (Cl_2_-Tyr) was developed based on the previously reported method [44]. Assay with ^13^C_6_-3-chloro-L-tyrosine (^13^C_6_-Cl-Tyr), and ^13^C_9_,^15^N-3,5-dichloro-L-tyrosine (^13^C_9_,^15^N-Cl_2_-Tyr) as an internal standard was linear (5–500 ng/mL for Cl-Tyr and 2.5–500 for Cl_2_-Tyr) with coefficient of regression, R^2^ > 0.99. Study samples (homogenized C57BL/6 lungs) were hydrolyzed with pronase and purified employing solid phase extraction as described previously [44]. Extracted samples were analyzed for Cl-Tyr and Cl_2_-Tyr. The results are reported as ng of Cl-Tyr and Cl_2_-Tyr/mg of protein.

### 4.9. Determination of BALF Inflammasome-relevant Cytokine Levels

Levels of the proinflammatory cytokine High Mobility Group Box 1 (HMGB1), which is released in response to inflammasome activation by a toxicant, Il-1β and TNFα were determined in BALF after 24 h of challenge as described previously [36,46]. For this, we used enzyme-linked immunosorbent assays (ELISA) using a commercially available ELISA kit (Chondrex Inc., Redmond, WA, USA). Samples were run undiluted in triplicate, and assays were performed according to manufacturer’s instructions. Levels of HMGB1 released into the BALF are reported as nanograms per milliliter (ng/mL) and levels of IL-1β and TNFα are reported as picograms per milliliter (pg/mL).

### 4.10. Western Blot Analysis

Immunoblot analysis of murine lung tissue at 24 h post-CuO-NP exposure was performed as previously described using a primary polyclonal antibody that recognizes 3-chlorotyrosine specific protein adducts (Hycult Biotech Inc., Wayne, PA, USA) [35,38,73]. Protein levels were quantified by densitometric analysis with β-actin normalization of protein expression using Gel-Pro Analyzer software (Version 6.0, MediaCybernetics, Silver Spring, MD, USA).

### 4.11. Isolation of Mouse Bone Marrow Neutrophils

Bone marrow neutrophils were derived using an established protocol [74]. Briefly, mice were euthanized, femur and tibia removed and placed in a petri dish containing ice-cold RPMI 1640 1× supplemented with 10% FBS and 1% penicillin/streptomycin, rinsed with 70% ethanol and epiphyses cut off. Bone marrow cells were flushed from both ends with RPMI supplemented with 10% FBS. EDTA was added and centrifuged followed by red blood cell lysis and repeated centrifugation. Neutrophils were separated by density gradient centrifugation using Histopaque 1119 and 1077 (MilliporeSigma, Burlington, MA, USA). Neutrophil number and viability were determined.

### 4.12. Determination of MPO-Dependent ACS Generation

CuO-NP-induced generation of MPO-dependent ACS was determined as previously described [30]. Briefly, purified mouse MPO (R & D systems, Minneapolis, MN, USA) or bone marrow-derived mouse neutrophils (10,000 cells/200 µL medium/well) in PBS, pH 7.4 (DPBS) with APF were exposed to 0, 10, or 20 µg CuO-NP. To determine MPO-dependent generation of ACS, experiments were performed in presence of 4-aminobenzoic acid hydrazide (100 µM) and the fluorescence of fluorescein, formed under HOCl-mediated cleavage of APF (5 µM), was determined. The fluorescence intensity was measured at excitation/emission wavelengths of 490 nm/515 nm in a Molecular Devices Spectramax i3 (Molecular Devices, Sunnyvale, CA, USA).

### 4.13. Statistical Analysis of the Data

Results are presented as means ± the standard error of the mean (SEM). All data were analyzed using two-way analysis of variance (ANOVA) to test for the main effects of CuO-NP exposure and LGM2605 treatment, and the interaction between these variables, on study outcome measures. If the overall F-test was statistically significant, Tukey’s HSD post hoc tests were conducted to determine significant differences between nanoparticle exposure groups and among treatment groups (no LGM2605 versus LGM2605). Statistical analyses were performed using GraphPad Prism version 6.00 for Windows, GraphPad Software, La Jolla, CA, USA, www.graphpad.com. Statistically significant differences were determined with *p*-value < 0.05. Asterisks shown in figures indicate significant differences between nanoparticle exposure groups (no exposure versus M120 versus CuO-NP) (* *p* < 0.05, ** *p* < 0.01, *** *p* < 0.001 and **** *p* < 0.0001). # shown in figures indicate significant differences between treatment groups (no LGM2605 versus LGM2605) (# *p* < 0.05, ## *p* < 0.01, ### *p* < 0.001 and #### *p* < 0.0001).

## 5. Conclusions

In summary, CuO-NP exposure in vivo resulted in chlorination toxicity in murine lung and treatment with LGM2605 either preventively or therapeutically, led to significant reductions in the effects of CuO-NP exposure. For the first time, we show that CuO-NP exposure results in chlorination damage in murine lung. Specifically, LGM2605 has a significant protective (anti-inflammatory) effect on CuO-NP-induced lung inflammatory cell population changes, lung injury, as well as lung protein chlorination toxicity in mice.
